# Molecular phylogenomics of the tribe Shoreeae (Dipterocarpaceae) using whole plastid genomes

**DOI:** 10.1093/aob/mcy220

**Published:** 2018-12-12

**Authors:** Jacqueline Heckenhauer, Ovidiu Paun, Mark W Chase, Peter S Ashton, A S Kamariah, Rosabelle Samuel

**Affiliations:** 1 University of Vienna, Department of Botany and Biodiversity Research, Vienna, Austria; 2 Senckenberg Research Institute and Natural History Museum Frankfurt, Frankfurt am Main, Germany; 3 Jodrell Laboratory, Royal Botanic Gardens, Kew, Richmond, UK; 4 Department of Environment and Agriculture, Curtin University, Bently, WA, Australia; 5 Harvard University, Department of Organismic and Evolutionary Biology, Cambridge, MA, USA; 6 University of Brunei Darussalam, Environmental and Life Sciences, Faculty of Science, Gadong, Brunei Darussalam

**Keywords:** Plastid genomes, Dipterocarpaceae, genome skimming, *Hopea*, next-generation sequencing, *Parashorea*, phylogenomics, *Shorea*, Shoreeae

## Abstract

**Background and Aims:**

Phylogenetic relationships within tribe Shoreeae, containing the main elements of tropical forests in Southeast Asia, present a long-standing problem in the systematics of Dipterocarpaceae. Sequencing whole plastomes using next-generation sequencing- (NGS) based genome skimming is increasingly employed for investigating phylogenetic relationships of plants. Here, the usefulness of complete plastid genome sequences in resolving phylogenetic relationships within Shoreeae is evaluated.

**Methods:**

A pipeline to obtain alignments of whole plastid genome sequences across individuals with different amounts of available data is presented. In total, 48 individuals, representing 37 species and four genera of the ecologically and economically important tribe Shoreeae *sensu* Ashton, were investigated. Phylogenetic trees were reconstructed using maximum parsimony, maximum likelihood and Bayesian inference.

**Key Results:**

Here, the first fully sequenced plastid genomes for the tribe Shoreeae are presented. Their size, GC content and gene order are comparable with those of other members of Malvales. Phylogenomic analyses demonstrate that whole plastid genomes are useful for inferring phylogenetic relationships among genera and groups of *Shorea* (Shoreeae) but fail to provide well-supported phylogenetic relationships among some of the most closely related species. Discordance in placement of *Parashorea* was observed between phylogenetic trees obtained from plastome analyses and those obtained from nuclear single nucleotide polymorphism (SNP) data sets identified in restriction-site associated sequencing (RADseq).

**Conclusions:**

Phylogenomic analyses of the entire plastid genomes are useful for inferring phylogenetic relationships at lower taxonomic levels, but are not sufficient for detailed phylogenetic reconstructions of closely related species groups in Shoreeae. Discordance in placement of *Parashorea* was further investigated for evidence of ancient hybridization.

## INTRODUCTION

Tribe Shoreeae of subfamily Dipterocarpoideae (Dipterocarpaceae, Malvales) is distributed in Southeast Asia, with its greatest diversity on Borneo (Ashton, 1988). Members of this ecologically important tribe often dominate the canopy of lowland forests. In addition to being major sources of commercial hardwood (Ashton, 2004), several species are valuable for non-timber products, such as their resins, nuts (butter fats) and tannins (Shiva and Jantan, 1998), and thus they are of great economic value.

Phylogenetic relationships in this tribe present a long-standing problem in the systematics of Dipterocarpaceae, and the existing classification is based mainly on morphological characters. According to Ashton, who monographed Dipterocarpaceae for *Flora Malesiana* (1982), tribe Shoreeae comprise five genera: *Dryobalanops* C.F.Gaertn., *Hopea* Roxb., the monotypic *Neobalanocarpus* P.S.Ashton, *Parashorea* Kurz and *Shorea* Roxb. ex C.F.Gaertn., the last including approx. 360 species. Generic limits in this tribe, especially that of *Shorea*, have been widely discussed. Based on floral characters, bark morphology and wood anatomy, Heim (1892) and Symington (1943) proposed groups of *Shorea* species. Classifications of Maury (1978) and Ashton (1982) are based on Symington’s (1943) four *Shorea* groups: selangan batu/balau, damar hitam (yellow meranti), meranti pa’ang (white meranti) and red meranti. Based on Maury’s (1978) studies of embryology in several species, *Shorea sensu*Ashton (1982) consists of the following six genera: *Shorea* (= selangan batu/balau), *Rubroshorea* (= red meranti) and *Richetia* F. Heim (= yellow meranti) in subtribe Shoreinae, and *Doona* Thwaites, *Pentacme* (DC.) P.S.Ashton and *Anthoshorea* Pierre (= white meranti) in subtribe Anthoshoreinae. Ashton (1982) maintained a single genus *Shorea sensu lato* (*s.l.*) solely based on sepal length in fruits. Mainly based on androecium, but also considering bark and wood anatomy, he recognized infrageneric taxa, i.e. 11 sections and eight subsections (Table 1).

**Table 1.** Comparative classifications of the tribe Shoreeae according to Maury (1978) and Ashton (1982)

Maury (1978)	Ashton (1982)
**Subtribe Shoreinae**	
Genus Shorea*	*Genus Shorea*
Section *Shoreae*	Section Shorea*
Section *Barbatae*	Subsection *Shorea*
	Subsection *Barbata*
	Section *Neohopea*
*Genus Richetia* ^†^	Section *Richetioides*^†^
Section *Richetioides*	Subsection *Richetioides*
	Subsection *Polyandrae*
Section *Maximae*	
*Genus Rubroshorea* ^‡^	
Section *Mutica*	Section *Mutica*^†^
Subsection *Mutica*	Subsection *Mutica*
Subsection *Auriculatae*	Subsection *Auriculatae*
Section *Ovalis*	Section *Ovalis*^‡^
Section *Neohopea*	Section *Neohopea*^‡^
Section *Rubella*	Section *Rubella*^‡^
Section *Brachypterae*	Section *Brachypterae*^‡^
Subsection *Brachypterae*	Subsection *Brachypterae*
Subsection *Smithianeae*	Subsection *Smithiana*
Section *Pachycarpae*	Section *Pachycarpae*^‡^
**Subtribe Anthoshoreinae**	
*Genus Anthoshorea* ^§^	Section *Anthoshorea*^§^
Section *Anthoshoreae*	Section *Doona*
*Genus Doona*	Section *Pentacme*
*Genus Pentacme*	
**Subtribe Parashoreinae**	
*Genus Parashorea*	*Genus Parashorea*
	
*Genus Hopea*
	Section *Hopea*
	Subsection *Hopea*
	Subsection *Pierra*
	Section *Dryobalanoides*
	Subsection. *Dryobalanoides*
	Subsection. *Sphaerocarpae*
	*Genus Neobalanocarpus*
	*Genus Dryobalanops*

*selangan batu/balau.

^†^yellow meranti.

^‡^red meranti.

^§^white meranti.

Previous molecular phylogenetic studies using only a few plastid markers (e.g. Gamage *et al.*, 2006; Heckenhauer *et al.*, 2017) or the nuclear *PgiC* gene (Kamiya *et al.*, 2005) indicated that *Shorea* groups *Anthoshorea* and *Doona* are more closely related to *Hopea* and *Neobalanocarpus* than to *Shorea* groups *Rubroshorea*, *Richetia* and *Shorea* (selangan batu/balau). The last form a clade with *Parashorea*, resulting in paraphyly of *Shorea sensu*Ashton (1982). The position of *Parashorea* and relationships within the genera of Shoreeae remain poorly to moderately supported in earlier studies (Gamage *et al.*, 2006; Heckenhauer *et al.*, 2017).

Facilitated by next-generation sequencing (NGS) techniques, genomic data are increasingly incorporated to investigate phylogenetic relationships, and restriction site-associated DNA sequencing (RADseq; Miller *et al.*, 2007; Baird *et al.*, 2008; Rowe *et al.*, 2011) has been successfully used to resolve taxonomic uncertainties within Shoreeae (Heckenhauer *et al.*, 2018). Besides RADseq, sequencing whole plastomes using NGS-based genome skimming has been employed in phylogenetic studies on plant families (Straub *et al.*, 2012). Because of its low mutation rates, high copy numbers and uniparental inheritance (in most seed plants, Birky, 1995), the gene-rich plastid genome has been widely used for inferring phylogenetic relationships among the major clades of green plants (Ruhfel *et al.*, 2014), angiosperms (Jansen *et al.*, 2007; Yang *et al.*, 2014), monocots (Barret *et al.*, 2016) and eudicots (Moore *et al.*, 2010), as well as at the level of closely related species (e.g. Ku *et al.*, 2013; Ma *et al.*, 2014; Malé *et al.*, 2014; Turner *et al.*, 2016; Bernhardt *et al.*, 2017; Fu *et al.*, 2017; S. D. Zhang *et al.*, 2017). Two complete plastid genomes have previously been assembled and annotated for *Vatica* L. of tribe Dipterocarpeae [*Vatica odorata* (Griff.) Symington (Cvetkovic *et al*., 2016), GenBank accession no. KX966283; and *Vatica mangachapoi* Blanco (Wang *et al.*, 2018), GenBank accession no. MH716496]. So far, no attempt has been made to use phylogenomic studies based on whole plastid genomes for the family Dipterocarpaceae.

Here, we explore with phylogenomic analyses information from 48 plastid genomes of 37 species representing four genera of Shoreeae *sensu* Ashton. We evaluate if phylogenetic inferences from plastome data are congruent to those obtained in previous studies using fewer plastid markers as well as RADseq-derived nuclear single nucleotide polymorphisms (SNPs). Our study provides genetic estimates of resources available for future research on this economically important family.

## MATERIALS AND METHODS

### Plant material and DNA isolation

Leaf material was collected, cleaned with a sponge of phyllosphere contaminants and stored in silica gel in the tropical forests of Brunei Darussalam and Sri Lanka (Supplementary Data Table S1). We included here 48 accessions, corresponding to 37 species: *Parashorea* (two species), *Hopea* (nine species), *Shorea sensu* Ashton (25 species) and *Dryobalanops* (one species). Of the 11 sections and eight subsections in the species-rich genus *Shorea* reported by Ashton (1982), nine sections and seven subsections are included in this study. The accessions included here represent five of the six genera (*Doona*, *Anthoshorea*, *Shorea*, *Richetia* and *Rubroshorea*) *sensu*Maury (1978).

Total genomic DNA was extracted using a modified sorbitol/high-salt cetyltrimethylammonium bromide (CTAB) protocol (Barfuss *et al.*, 2016) from approx. 40 mg of silica gel-dried tissue (Chase and Hills, 1991). The DNA content was quantified using the Qubit 3.0 Fluorometer with the dsDNA HS Assay Kit (Thermofisher).

### Library preparation

Two NGS libraries with 24 individuals each were prepared. Targeting an average fragment size of 350 bp, 500 ng of DNA was sheared by sonication using a Bioruptor Pico (Diagenode, Belgium) with seven cycles of 15 s ‘on’ and 90 s ‘off’ at 6 °C. Aiming for an even coverage across the length of the plastomes, library preparation was performed using the TruSeq DNA PCR-Free Low Throughput Library Prep Kit (Illumina, USA), including indexed adapters from the TruSeq DNA CD Indexes (Illumina) according to the manufacturer’s protocol. Individual libraries were pooled, targeting an equal representation of each individual in the final libraries. Both libraries were sequenced on an Illumina HiSeq 2500 at VBCF Vienna NGS Unit (http://vbcf.ac.at/ngs/) as 125 bp paired-end reads. All generated genomic data are deposited as a BioProject at the NCBI Sequence Read Archive (BioProject ID PRJNA419625, SRA Study ID SRP142704; SRA accessions for each sample are given in Supplementary Data Table S1).

### Plastid genome assembly and annotation

Demultiplexing of the raw data was performed based on index reads, allowing for a maximum of one mismatch using the Picard BamIndexDecoder (included in the Picard Illumina2bam package; available online at: https://github.com/wtsi-npg/illumina2bam). The number and quality of raw reads obtained from each individual were evaluated with FASTQC version 0.11.5 (Andrews, 2010). The plastid genome of *Hopea dryobalanoides* Miq. (accession: 12-4150) was assembled with the assembly pipeline FAST-PLAST version 1.2.7 (available online at: https://github.com/mrmckain/Fast-Plast) using Malvales in the - -bowtie_index option. The assembled plastome was validated by mapping back the reads of the same accession using the CLC GENOMICS WORKBENCH version 8.0 (Qiagen, Germany) with default settings. An even coverage was expected as PCR-free kits have been used for library preparation (see above). However, the junctions between the single copy regions and the inverted repeats presented drops in coverage. Based on the mappings, the assembly was manually corrected, and mapping was repeated until coverage remained uniform across the entire plastid genome. We attempted to assemble the plastid genomes of several individuals with the same pipeline.

**Fig. 1.**
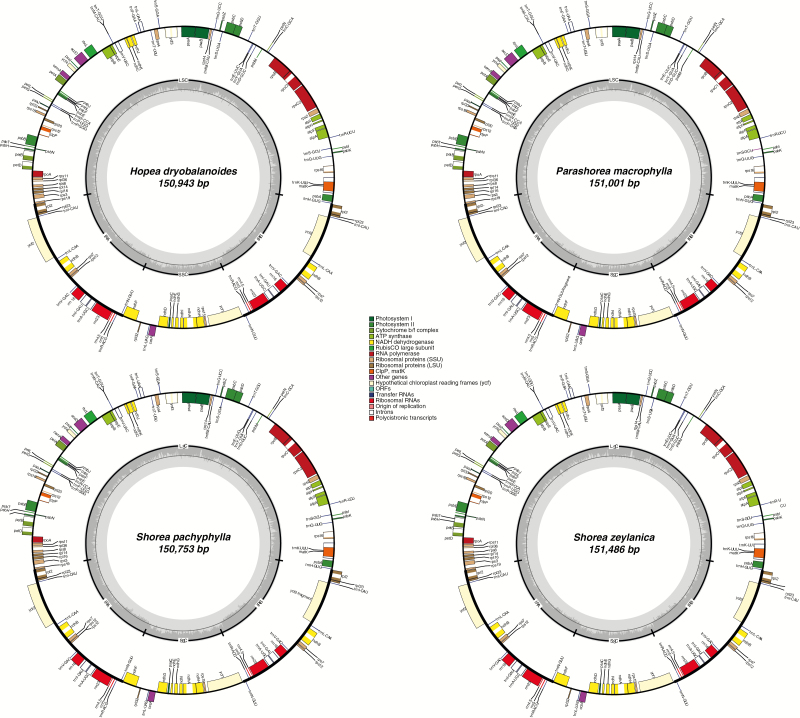
Graphic representation of the annotated plastid genomes of selected species of tribe Shoreeae.

Plastome annotations of representative species, i.e. *Hopea dryobalanoides* (GenBank accession no. MH791329), *Parashorea tomentella* (GenBank accession no. MH791330), *Shorea pachyphylla* (GenBank accession no. MH841940) and *Shorea zeylanica* (GenBank accession no. MH841939), were performed online using GeSeq (Tillich *et al.*, 2017) under activation of the ‘MPI-MP chloroplast reference set’ which includes annotation of ‘CDS’ (protein-coding regions) and rRNAs. ARAGORN version 1.2.38 (Laslett and Canback, 2004) was used to annotate tRNAs. GenBank files of the order Malvales were then selected from the ‘Server Reference’ menu. Resulting sequence features, the sequence itself and its six-frame translation were visualized with the annotation tool ARTEMIS (Rutherford *et al.*, 2000) and annotations were edited manually. Circular plastid genome maps (Fig. 1) were visualized with OGDRAW version 1.2 (Lohse *et al.*, 2007).

For some individuals, the number of reads appeared insufficient for a complete *de novo* assembly of the whole plastid genome, and the assemblies obtained were fragmented.

Therefore, we developed a mapping-based pipeline to extract whole plastid genome sequence information across all accessions. First, the demultiplexed reads were quality-filtered using the program TRIMMOMATIC v.0.33 (Bolger *et al.*, 2014). Illumina adapter sequences were removed with the ILLUMINACLIP option allowing for a maximum of two mismatches, a palindrome clip threshold of 30 and a ten simple clip threshold. Simultaneously, bases with a quality lower than 13 were removed from the beginning and end of reads. The reads were also scanned with a four base wide sliding window, removing them when average quality per base dropped below 15. Finally, reads shorter than 36 bases long were excluded.

A BWA (Li and Durbin, 2010) index for the *de novo* assembled reference plastome of *H. dryobalanoides* (accession 12-4150) was created using the linear-time (ls) algorithm for constructing the suffix array recommended for small genomes. After generating a fasta file index using the faidx option of SAMtools v. 1.4 (Li *et al.*, 2009), a sequence dictionary for the reference was built using Picard tools v.2.9.0 (Wysoker *et al.*, 2013). The filtered reads of each accession were then mapped to the reference with BWA-MEM. The option -M was applied to flag as secondary shorter split hits. The resulting aligned SAM files were sorted by co-ordinate, and read groups were added to resulting bam files using Picard tools. Realignments around indels were performed with the Genome Analysis Toolkit (GATK; McKenna *et al.*, 2010) version 3.7.0, thinning data to a maximum of 100 000 reads per interval. Calling of SNPs and indels was conducted via local *de novo* assembly of haplotypes using the GATK HaplotypeCaller in the ERC GVCF mode with a sample ploidy of 1 without application of a specialized PCR error model. Joint genotyping was performed on the resulting gVCF files with the GenotypeGVCFs tool of GATK. To avoid complications related to aligning indel-rich regions, and as indels themselves are usually not used in phylogenetic reconstruction (e.g. Turner *et al.*, 2016; M. Y. Zhang *et al.*, 2017; Lu *et al.*, 2018; Tosso *et al.*, 2018), only substitution information has been retained in the final vcf file using the -selectType SNP option in GATK following Balao *et al.* (2017). Finally, the FastaAlternateReferenceMaker tool in GATK was used to replace the SNP information in the reference, resulting in a fasta file for a synthetic sequence of the plastid genome of each accession. The fasta files of each individual were further concatenated into a single data matrix, which resulted in a fully aligned fasta file.

### Alignment and phylogenetic analyses

Based on a previous study (Heckenhauer *et al.*, 2017), the plastid genome sequence of *V. odorata* of the sister tribe Dipterocarpeae was used as outgroup. The sequence was downloaded from GenBank (accession no. KX966283). Because the single-copy region (SSC) of *V. odorata* KX966283 was found to be a reverse complement of that in the species included in our study and in most other angiosperms (see, for example, Palmer, 1983; Liu *et al.*, 2013; Cai *et al.*, 2015, Lu *et al.*, 2018), it was complemented and inverted using Geneious version 8.0.5 (Kearse *et al.*, 2012), before aligning it to the ingroup sequences with default settings in MAFFT version 7.22 (available online at: http://mafft.cbrc.jp/alignment/server/). The alignment was inspected in BIOEDIT version 7.0.4 (Hall, 1999). For the phylogenetic analyses, only one copy of the inverted repeat was included in the final alignment, leading to a matrix of 130 649 characters.

A maximum parsimony (MP) analysis was performed in PAUP version 4.0a149 (Swofford, 2016) via heuristic search with stepwise addition, 1000 replicates of random addition sequence and tree bisection–reconnection (TBR) branch swapping. Clade support was estimated by bootstrapping (Felsenstein, 1985) with 1000 replicates of heuristic search (as above).

A maximum likelihood (ML) analysis was conducted using RAxML version 8.2.4 (Stamatakis, 2014). Rapid bootstrap analyses (1000 replicates) with a subsequent ML search for the best scoring tree were conducted in a single program run. A fast inference under the general time-reversible (GTR) model of nucleotide substitution with optimization of substitution rates and GAMMA model of rate heterogeneity (i.e. the GTRGAMMA model) was executed as recommended for <50 taxa. GTR rate parameters were optimized using the Broyden–Fletcher–Goldfarb–Shanno (BFGS) method.

In addition, we conducted Bayesian inference (BI) using Mr. Bayes version 3.2.6 (Huelsenbeck and Ronquist, 2001; Ronquist and Huelsenbeck, 2003). The most complex substitution model, general-time reversible (GTR + I + GAMMA) model with six substitution types (one for each pair of nucleotides) and gamma-distributed rate variation across sites and a proportion of invariable sites was employed. Two independent Metropolis-coupled Markov chain Monte Carlo (MCMC) analysis each with 20 million generations, sampling each 1000th generation, were run. The initial 25 % of trees obtained from each MCMC run was removed as burn-in; the remaining trees of both runs were used to calculate the maximum clade credibility tree. The resulting trees were rooted with *Vatica* according to earlier results (Heckenhauer *et al*., 2017, 2018) and visualized with FigTree version 1.4 (available online at: http://tree.bio.ed.ac.uk/software/figtree/).

A phylogenetic network based on already available nuclear RADseq data (Heckenhauer *et al.* 2018) was produced in SPLITSTREE 4.10 (Huson and Bryant, 2006) to detect patterns of reticulation caused by hybridization. Splits trees were drawn using the uncorrected p method, and only individuals comprising the present study were included.

## RESULTS

The number of raw Illumina paired-end reads per individual after demultiplexing ranged from 2.2 to 24 million, with an average of 9.8 million (s.d. 4 million). However, only between 29 000 and 470 000 of these pairs of reads originated from the plastid genome (average 191 000 ± 118 000 s.d.) as indicated by mapping to the reference genome assembled in the next step.

### Characteristics of the plastid genomes

A total of 119 708 reads were mapped to the reference plastid genome of *Hopea dryobalanoides* (accession: 12-4150). The size of the plastid genome ranges from 150 669 bp in *Shorea albida* Symington ex A.V.Thomas to 152 479 bp in *Dryobalanops lanceolata* Burck. There were 131 genes, comprising 85 coding genes, 38 tRNA genes and eight rRNA genes (Fig. 1). In total, 114 of the 131 genes are single copy and 17 are duplicated in the inverted repeats. Specifically, all four rRNA genes, seven of the tRNA genes and six other coding genes are duplicated within the inverted repeats. The GC content averages 37 %.

### Phylogenetic analyses of the plastid genomes

Mapping the reads to the reference plastid genome resulted in a coverage that ranged from 20.2- to 369-fold (average 144 ± 93-fold) for each accession. Of the 130 649 characters included in the final matrix, 4979 characters were potentially parsimony informative. The MP analysis resulted in a single most parsimonious tree of 15 457 steps (results not shown), having a retention index of 0.89 and a consistency index of 0.78.

**Fig. 2.**
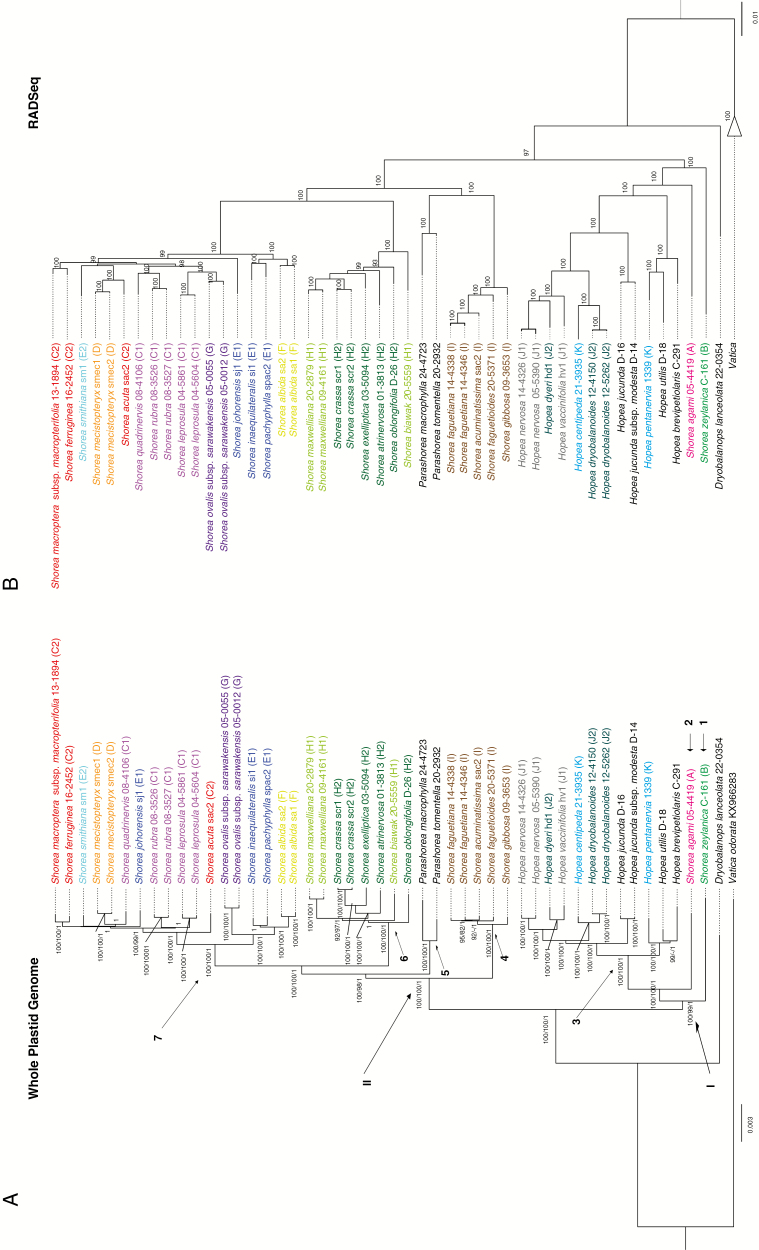
Phylogenetic trees resulting from the maximum likelihood analysis of (A) the plastome data set of the present study and (B) the SNP data set derived from RADseq in a previous study (Heckenhauer *et al.*, 2018). (A) Bootstrap percentages (≥90 %) from maximum parsimony and maximum likelihood analyses and posterior probabilities (BI >0.90) are given in this order. The two major clades (I and II) and the indicated genera/groups of *Shorea*: 1 *Doona*, 2 *Anthoshorea*, 3 *Hopea*, 4 *Richetia*, 5 *Parashorea*, 6 *Shorea* (selangan batu/balau) and 7 *Rubroshorea*. (B) For comparative reasons, the tree is pruned to species investigated in this study. Bootstrap percentages (≥90 %) from maximum likelihood analysis are given. Individuals are coloured according to different (sub-)sections. Sections and subsections according to Ashton are given: A, section *Anthoshorea*; B, section *Doona*; C, section *Mutica*; C1, subsection *Mutica*; C2, subsection *Auriculatae*; D, section *Pachycarpae*; E, section *Brachypterae*; E1, subsection *Brachypterae*; E2, subsection *Smithiana*; F, section *Rubella*; G, section *Ovalis*; H, section *Shorea*; H1, subsection *Barbata*; H2, subsection *Shorea*; I, section *Richetioides* subsection *Richetioides*; J, section *Dryobalanoides*; J1, subsection *Sphaerocarpae*; J2, *Dryobalanoides*; K, section *Hopea* subsection *Hopea*.

The best-scoring ML tree with bootstrap percentages (BPs) from the MP (BP_MP_) and ML (BP_ML_) analyses and posterior probabilities from the BI (PP) is shown (Fig. 2A). The topologies of the trees resulting from MP, ML and BI analyses were congruent. There are two highly supported major clades in the ingroup (Fig. 2A: I and II). The first main clade (Fig. 2A: I: BP_MP_ 100, BP_ML_ 99, PP 1.00; this order of support will be used throughout) contains *Doona* (1), *Anthoshorea* (2) and monophyletic *Hopea* (3). The second main clade (Fig. 2A: II: 100, 100, 1.00) has four monophyletic groups that correspond to *Richetia* (4), *Parashorea* (5), *Shorea* (selangan batu/balau; 6) and *Rubroshorea* (7). Here, *Rubroshorea* is sister to *Shorea* (selangan batu/balau, Fig. 2A: 100, 100, 1.00) followed by *Parashorea* (Fig. 2A: 100, 98, 1), and *Richetia* is sister to all three (Fig. 2A: 100, 100, 1.00). Regarding relationships within *Hopea* and *Shorea*, some sections of Ashton (1982) are not monophyletic (e.g. section *Mutica* of *Rubroshorea*, Fig. 2A). However, several terminal relationships between species within *Richetia*, *Shorea/Shorea* (selangan batu/balau) and *Rubroshorea* are weakly supported. The phylogenetic patterns obtained from a RADseq-derived SNP data set (Heckenhauer *et al.*, 2018) show differences in topology from those of the plastid data (Fig. 2A, B), with the main incongruence being the position of *Parashorea*. However, a SplitsTree phylogenetic networks based on the nuclear RADseq data from a previous study did not reveal any significant reticulation pattern (potentially caused by hybridization) involving *Parashorea* (Supplementary Data Fig. S2).

## DISCUSSION

### Plastid genome structure and comparisons

The plastid genomes presented in this study are the first fully sequenced plastid genomes of Shoreeae reported in the literature, providing insights into the characteristics of plastid genomes of members of this tribe. Having a size range of 150 669–152 479 bp, the genome size in Shoreeae is relatively small. Although a similar size has been reported for *Vatica odorata* (151 465 bp; Cvetkovic *et al*., 2016) and *Vatica mangachapoi* (151 538 bp; Wang *et al.*, 2018), other species in the order Malvales have slightly larger plastomes ranging from 159 039 bp in *Gossypium stocksii* Mast. (GenBank accession no. JF317355, Malvaceae) to 174 885 bp in *Aquilaria yunnanensis* S.C.Huang (Thymelaceae, Zhang *et al.*, 2018). The gene content and order of the studied plastomes are comparable with those of other members of Malvales. The GC content of 37 % is consistent with those observed in other Malvales [e.g. *Durio zibethinus* L. (Cheon *et al.*, 2017), *Gossypium* L. (Lee *et al.*, 2006), *Theobroma cacao* L. (Kane *et al.*, 2012) and *V. odorata* (Cvetkovic *et al*., 2016)].

### Phylogenetic analyses of the plastid genomes

The APG system (Angiosperm Phylogeny Group, 1998, 2003, 2009, 2016), a mostly molecular-based classification, is a great example of how molecular approaches have enhanced our understanding of phylogeny and plant systematics in the last two decades. Although traditional Sanger sequencing is limited to a small number of loci, NGS approaches provide much larger data sets, up to whole genomes including mitochondrial, nuclear and plastid DNA, and thus have become widely used in plant systematics (Straub *et al.*, 2012). However, in plants, the mitochondrial genome exhibits a complex structure with many repeated regions that make assembly difficult (Malé *et al.*, 2014), and only a few studies have focused on complete mitochondrial DNA genomes (e.g. Notsu *et al.*, 2002; Yu *et al.*, 2018). Previous studies have revealed only low levels of variation in plant mitochondrial DNA genomes (Malé *et al.*, 2014). In spite of being highly variable, analyses of the nuclear ribosomal region have led to poorly resolved trees in earlier studies (Malé *et al.*, 2014) and could not provide well-supported phylogenetic relationships among closely related species (Turner *et al.*, 2016). Therefore, we did not attempt to assemble either mitochondrial genomes or nuclear rDNA, but instead focused on the usefulness of complete plastid genomes to resolve phylogenetic relationships in these non-model organisms (Dipterocarpaceae) across a relatively wide taxonomic scale (tribal level).

Systematics of Dipterocarpaceae have been widely discussed, and several molecular studies, mostly of single molecular markers, have been carried out in the past (e.g. Kamiya *et al.*, 1998; Dayanandan *et al.*, 1999; Gamage *et al*., 2003, 2006).

The topologies of the phylogenetic trees obtained based on the whole plastid genomes (130 649 bp) are similar to those revealed in a previous study based on three plastid regions (5911 bp; Supplementary Data Fig. S1; Heckenhauer *et al.*, 2017). However, analysing whole plastid genomes provided stronger support for the main clades of genera and groups of *Shorea*. The two monophyletic genera *Hopea* (Fig. 2A; 3) and *Parashorea* (Fig. 2A; 5) appear to be nested within the genus *Shorea sensu* Ashton. Thus, genus *Shorea sensu* Ashton is not monophyletic and is divided into five major groups. These correspond to earlier morphological classifications of Maury (1978): *Anthoshorea* (1), *Doona* (2), *Richetia* (4) *Shorea* (6) and *Rubroshorea* (7; Fig. 2A). Results of this study clearly show that using the whole plastid genome is useful for resolving phylogenetic relationships among the major groups within Shoreeae: *Hopea*, *Parashorea* and the five groups of *Shorea sensu* Ashton, *Doona*, *Anthoshorea*, *Richetia*, *Shorea* (selangan batu/balau) and *Rubroshorea*. The position of *Parashorea* had been unclear in previous investigations (Supplementary Data Fig. S1; Heckenhauer *et al.*, 2017), but its position as sister to *Rubroshorea* and *Shorea* (selangan batu/balau) is highly supported here (Fig. 2A). Species relationships within these main clades still remain unresolved and weakly supported due to low levels of variation (Fig. 2A; in *Rubroshorea*, *Shorea* and *Richetia*). Similar results have been observed in *Diospyros*, Ebenaceae (Turner *et al.*, 2016). Higher support and better resolution for the terminal branches were obtained in RADseq-derived phylogenetic trees (Fig. 2B; Heckenhauer *et al.*, 2018).

In this study, we also compared phylogenetic relationships from plastome data with those obtained from nuclear RADseq-derived SNPs (Heckenhauer *et al.*, 2018). Phylogenetic trees obtained from RADseq-derived SNP data sets not only exhibit higher support and better resolution in the terminal branches but also highlight some differences in topologies (Fig. 2A, B). The main incongruence between the two phylogenetic trees is the position of *Parashorea*. In the plastome trees, *Parashorea* is sister to *Rubroshorea* and *Shorea* (selangan batu/balau), whereas in the RADseq trees *Parashorea* forms a clade with *Richetia*. These incongruent positions of *Parashorea* are highly supported in phylogenetic trees obtained from both plastome and RADseq data. In a previous investigation of genetic differentiation of Indonesian Dipterocarpaceae using amplified fragment length polymorphism (AFLP) markers, *Parashorea* clustered together with *Hopea* (Cao *et al.*, 2006). The observed discrepancy of *Parashorea* in the plastome and RADseq-derived tree suggests that the origin of this genus may be associated with ancient hybridization. Hybridization plays a major part in speciation and evolution of angiosperms (Stebbins, 1959; Rieseberg, 1997), and it has been reported in Dipterocarpaceae in the past (Bawa, 1998; Kamiya *et al.*, 2011). Using the RADseq-derived SNP data set (Heckenhauer *et al.*, 2018), we attempted to detect patterns of reticulation in *Parashorea* that could provide evidence of hybridization. Therefore, splits were drawn using the uncorrected p method in SPLITSTREE 4.10 (Huson and Bryant, 2006). However, there is no evidence for hybridization in the split tree (Supplementary Data Fig. S2). Another explanation for the phylogenetic incongruence in the position of *Parashorea* between different data sets could be incomplete lineage sorting. At this point, our results do not allow us to discuss these possible scenarios in detail since a more comprehensive taxon sampling within *Parashorea* would be required. It is also important to consider that plastid trees may not reflect the species tree (Rosenberg, 2002; Degnan and Rosenberg 2006). Including many independent markers from the whole genome, the RADseq-derived SNP data set (Heckenhauer *et al.*, 2018) represents more of the history recorded in these genomes and is thus considered as a much better estimation of the species tree in tribe Shoreeae. Nevertheless, our study provides important genetic resources for further studies of Dipterocarpaceae.

### Taxonomic implications

Because classifications within the tribe Shoreeae have been widely discussed, the results obtained in this study also provide important insights for morphology-based systematists. In this paragraph, we discuss the extent to which our results point towards revision of current (Ashton, 1982) and proposed (Maury, 1978) taxonomy. The following potential taxonomic revisions are supported from this investigation: first, exclude published infrageneric categories (sections and subsections) in *Shorea* (selangan batu/balau) and *Rubroshorea* since most of these are not monophyletic in the molecular studies (Heckenhauer *et al*., 2017, 2018). Secondly, either expand *Shorea* to include all of these (*Shorea s.l.*) or recognize *Doona*, *Anthoshorea*, *Richetioides* and *Rubroshorea* as genera, reducing *Shorea* (selangan batu/balau) to just *Shorea sensu stricto.* However, to use these molecular groupings to develop a new taxonomic scheme, diagnostic morphological or anatomical characters need to be recognized. This will be difficult due to many aspects: the single character of the number of sepals in fruit (long vs. short) used in Ashton’s circumscription of the genus *Shorea* is not suitable given the fact that several *Shorea* species exhibit only short sub-equal sepals. Defining adequate characters is especially challenging for *Rubroshorea*. Even though Symington (1943) and later authors (Maury, 1978; Ashton, 1982) recognized infrageneric taxa based on characters of androecium and bark morphology, supported by anatomy, there is no single diagnostic character for *Rubroshorea.* It is consistently held together by its red colour wood, a character that also occurs in other taxa, e.g. *Shorea guiso* (Blco) Bl. of Ashton’s section and subsection *Shorea.* The clear and consistent distinctions on the independent diagnostic characters of androecium and bark must eventually be shown to have a genetic base. Given the economic and ecological importance of tribe Shoreeae, the molecular phylogenetic studies must now prompt a search for diagnostic characters, a necessary prerequisite for any systematic and taxonomic revision.

## SUPPLEMENTARY DATA

Supplementary data are available online at https://academic.oup.com/aob and consist of the following. Figure S1: best scoring maximum likelihood tree obtained from six plastid DNA regions in a previous study. Figure S2: SplitsTreeNetwork (NeighborNet) based on uncorrected p distance derived from a RADseq-derived data set obtained in a previous study. Table S1: samples used in this study.

mcy220_suppl_Figure_S1

mcy220_suppl_Figure_S2

mcy220_suppl_Table_S1
