# Supplementary figures and images for: Molecular phylogenomics of the tribe Shoreeae (Dipterocarpaceae) using whole plastid genomes
Source: Ann Bot. 2018 Dec 12;123(5):857–65. doi: 10.1093/aob/mcy220 (PMC6526321; doi:10.1093/aob/mcy220)

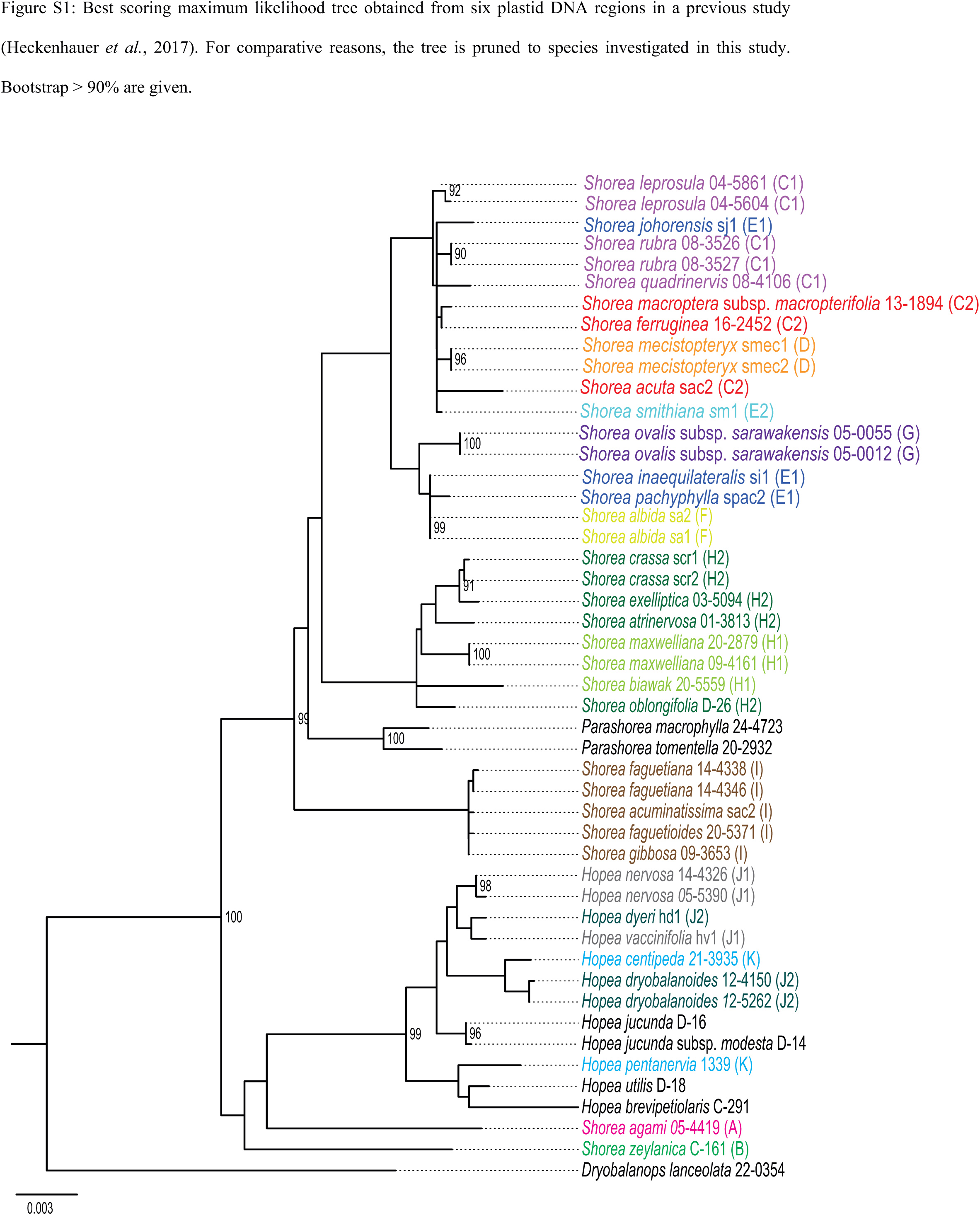

Supplement: mcy220_suppl_Figure_S1 [file mcy220_suppl_figure_s1.jpeg]

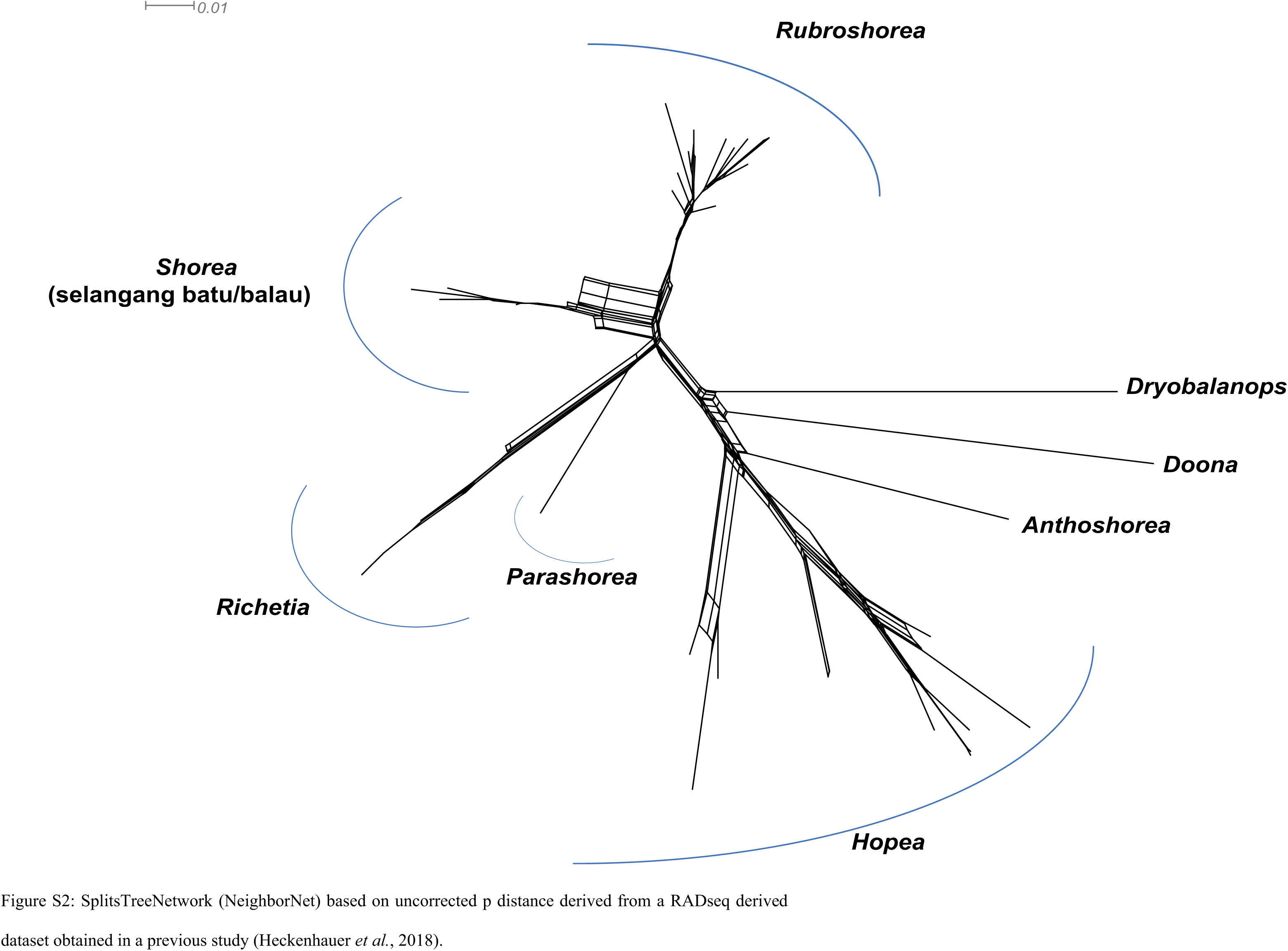

Supplement: mcy220_suppl_Figure_S2 [file mcy220_suppl_figure_s2.jpeg]
